# Persistent Bradycardia with the Long-term Use of Phenytoin and Oxycodone: First Case Report

**DOI:** 10.7759/cureus.2169

**Published:** 2018-02-08

**Authors:** Intekhab Askari Syed, Aaron R Kuzel, Muhammad Uzair Lodhi, Waliul Chowdhury, Umar Rahim, Mustafa Rahim

**Affiliations:** 1 Medical Student, Department of Medicine, Raleigh General Hospital, Beckley, Wv; 2 Department of Emergency Medicine, Lincoln Memorial University-Debusk College of Osteopathic Medicine; 3 Pre-Medical Student, Department of Sciences, Queens University of Charlotte, Nc; 4 Assistant Clinical Professor of Internal Medicine, West Virginia University School of Medicine

**Keywords:** persistent bradycardia, phenytoin toxicity, oxycodone overdose

## Abstract

Phenytoin is a medication that is used primarily in the treatment of epilepsy as well as generalized tonic-clonic seizures and status epilepticus. Phenytoin is also considered a class IB antiarrhythmic medication by shortening the duration of the action potential and increasing myocardial conduction. The neurologic adverse effects of phenytoin are well-documented and include altered mental status, ataxia, and nystagmus. Some adverse cardiac manifestations have also been reported, including arrhythmias, hypotension, and respiratory arrest. Oxycodone is an opioid that exerts its effects by binding to Mu opioid receptors located in the central nervous system. This selective binding results in the opening of potassium (k+) channels and the closing of calcium channels, decreasing synaptic transmission. Oxycodone, unlike phenytoin, has not been observed to elicit cardiotoxicity independent of other medications. However, in combination with other medications, bradycardia and hypotension have been observed. We report the case of a 62-year-old male who developed persistent bradycardia following treatment with phenytoin and oxycodone for seizure disorder and pain, respectively. To our knowledge, this is the first case report where bradycardia was induced by a combination of these medications.

## Introduction

Oxycodone and phenytoin are both well known for their cardiotoxic side-effect profile, especially at higher doses [1-2]. The oral administration of phenytoin is particularly prone to neurological manifestations [1]. A few studies have been reported to cause bradycardia following the toxicity of phenytoin. On the other hand, it is also common for oxycodone to cause bradycardia at higher doses [2]. Furthermore, drug-drug interactions could be a factor behind idiopathic bradycardia manifestations. There has not been any study established to date for oxycodone-induced bradycardia. Thus, we will be presenting persistent bradycardia with normal hemodynamics in a patient with long-term phenytoin and oxycodone use.

## Case presentation

A 62-year-old Caucasian male smoker with a past medical history of acute and chronic obstructive pulmonary disease, coronary artery disease, seizure disorder, deep vein thrombosis, anxiety, and phenytoin toxicity presented to the emergency department complaining of upper abdominal pain that started a few days ago. He stated the severity of his pain was moderate without radiation and without relieving or aggravating factors. He denies fever or chills. He denied any recent injury, trauma, dysuria, hematuria, coughing, congestion, rashes, nausea, vomiting, chest pain, shortness of breath, dysphagia, odynophagia, heartburn, or reflex symptoms. The patient further denied any history of thyroid disease, diabetes, excessive bleeding, easy bruising, color changes, or hyperpigmentation of the skin. His current medications include warfarin (4 mg), phenytoin (300 mg), and oxycodone (20 mg).

Upon examination, the patient was resting comfortably and did not appear to be in any acute distress. His vital signs were as follows: blood pressure of 136/90 mm Hg, heart rate of 43 beats per minute (bps), respiratory rate of 19 breaths per minute (bpm), and no fever. Upon abdominal examination, the patient exhibited abdominal tenderness on palpation without any rebound tenderness, guarding, or peritoneal signs. The rest of his examination findings were unremarkable.

**Figure 1:**
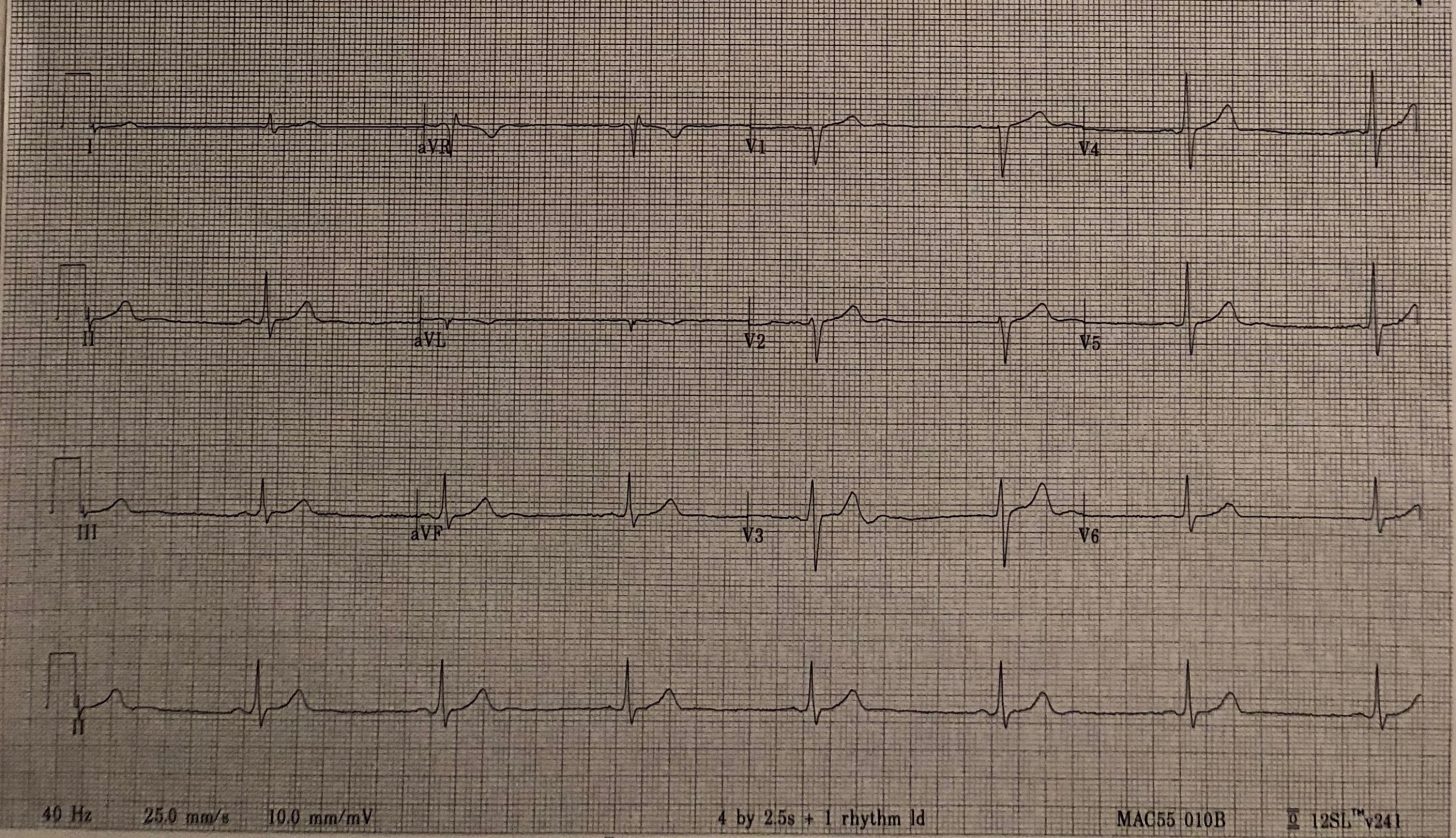
Showing marked sinus bradycardia

The patient was further evaluated for his persistent bradycardia. His warfarin was withheld until his levels of prothrombin time, partial thromboplastin time, along with international normalized ratio were assessed (Table 1). Serum phenytoin level (Table 2), complete blood test (Table 3), and complete metabolic panel (Table 4) were ordered. A diagnosis of phenytoin toxicity was established after ruling out the other possibilities, and the patient was assessed subsequently. It was found that the patient had multiple admissions for similar, prior illnesses. Electrocardiography (EKG) was done due to his continuous bradycardia and showed a sinus rhythm with long R-R intervals (Figure 1). The patient was also evaluated for his abdominal pain. Abdominal imaging, which included magnetic resonance cholangiopancreatography, was negative.

**Table 1:** Serum coagulation test

Prothrombin test and international normalized ratio		
Test	Result	Reference
Prothrombin test, PT	64.2 seconds	9.6-11.8 seconds
International normalized ratio, INR	6.65	0.89-1.12

**Table 2:** Serum phenytoin level

Serum phenytoin		
Test	Result	Reference
Phenytoin Level	32 mcg/mL	10-20 mcg/mL

**Table 3:** Complete blood count

Complete blood count		
Test	Result	Reference
White blood cell, WBC	6.7 k/mm3	6.3-9.1 k/mm3
Red blood cell, RBC	5.28 mil/mm	4.50-6.30 mil/mm
Hemoglobin, HBG	15.8 g/dl	12.1-15.9 g/dl
Hematocrit, HCT	49.80%	35.8-46.9%
Mean corpuscular volume, MCV	94 um3	80-100 um3
Platelets	158 k/mm3	140-440 k/mm3

**Table 4:** Complete metabolic panel

Complete metabolic panel		
Test	Result	Reference
Sodium, Na	138 mmol/L	137-144 mmol/L
Potassium, K	4.5 mmol/L	3.5-5.0 mmol/L
Chloride, Cl	105 mmol/L	98-108 mmol/L
Carbon dioxide, CO2	26 mmol/L	21-32 mmol/L
Anion gap	12 mmol/L	0-40 mmol/L
Glucose	99 mg/dL	70-110 mg/dL
Blood urea nitrogen, BUN	32 mg/dL	7-18 mg/dL
Creatinine, Cr	0.9 mg/dL	0.6-1.3 mg/dL
Calcium, Ca	8.3 mg/dL	8.5-10.1 mg/dL
Alkaline Phosphate, ALP	103 units/L	45-117 units/L
Lipase	72 units/L	73-393 units/L
Bilirubin	0-2 mg/dL	0.2-1.0 mg/dL
Aspartate transaminase, AST	16 units/L	15-37 units/L
Alanine transaminase, ALT	19 units/L	12-78 units/L

A plan was constructed to discontinue his phenytoin and re-evaluate the patient. The patient’s heart rate improved slightly but was still bradycardic (Table 5). It was thought that an additional factor could be playing a role in this mechanism. A further diagnosis was made of phenytoin- and oxycodone-induced bradycardia. After withholding his medication, his abdominal pain eventually resolved. Due to the patient’s chronic daily oxycodone use, it was decided the patient would remain on his normal dose to prevent withdrawal. He has been taking high-dose oxycodone because of concurrent phenytoin (P450 inducer) use. The patient was discharged on levetiracetam for his seizure disorder and his other home medications were continued.

**Table 5:** Serial vital signs

Serial vital signs				
Event	Blood pressure (mm Hg)	Pulse (bps)	Respiration (bpm)	Temperature (F)
Admission	136/90	43	19	98.2
Stopped phenytoin	132/68	54	19	98.2
Discharge	122/63	57	18	97.8

## Discussion

Phenytoin is a medication used in the treatment of epilepsy as well as generalized tonic-clonic seizures and status epilepticus. Phenytoin has minimal efficacy in absence, atonic, and myoclonic seizures. Phenytoin is favored when compared to benzodiazepines due to their limited sedative effects and repression of respiratory drive [3]. Phenytoin exerts its effect on cardiac sodium channels by blocking the inward movement of sodium in phase 0, preventing depolarization. This is represented on an EKG with a widening of the QRS complex [4]. This mechanism can thereby delay or suppress the atrioventricular nodal conduction that leads to bradyarrhythmia. However, phenytoin has almost no effect on interventricular conduction or the sinoatrial node [4]. Toxicity from phenytoin ingestion typically first manifests as neurological symptoms affecting the cerebellar and vestibular functions of the nervous system [1]. The neurological manifestations of phenytoin toxicity include altered mental status, ataxic gaits, and nystagmus [5]. Phenytoin toxicity is typically the result of a suicidal attempt via an intentional overdose, drug interactions, or elevated dosage adjustments [1]. We had no reason to believe this patient was intentionally overdosing on his medications.

Phenytoin is a class IB antiarrhythmic medication. It shortens the duration of the action potential and increases myocardial conduction [3]. In addition to neurologic manifestations, some serious adverse cardiac manifestations have been observed that include arrhythmias, hypotension, and respiratory arrest. While cardiotoxic effects have been well-documented in the use of intravenous phenytoin administration, these effects are not commonly seen in oral phenytoin administration [1,3].

In a study by Guldiken et al., 32 clinical trials and 10 case reports regarding phenytoin toxicity were examined. They determined that a rapid infusion of 50 mg/min or a greater dose of phenytoin was the major cause of mortality. However, of the 32 clinical trials, there were no reports of cardiovascular adverse effects leading to mortality. Of the 10 case reports, two of the 12 patients receiving an intravenous infusion of phenytoin developed bradycardia and asystole. In a third patient, apnea was the only sign prior to the patient’s death. After reviewing the studies, the authors concluded that the majority of patients who died from intravenous phenytoin administration had comorbid cardiac and metabolic disorders. They also determined that patients with a pre-existent cardiac disease were more vulnerable to intravenous phenytoin administration. In addition to this review, the authors also agreed that the oral administration of phenytoin was considered a significantly safer alternative to intravenous dosing [3].

Although uncommon, oral phenytoin has been documented to cause severe bradyarrhythmias and cardiovascular issues. In one case, severe hypotension and sinus bradycardia developed following the consumption of 20 g of phenytoin and 500 mg of glibenclamide in a suicide attempt. The authors suggested that this cardiotoxicity of phenytoin could be due to the patient’s metabolic disorder or interactions with the sulfonylurea he was using [1]. In another case, sinus node arrest with symptomatic junctional bradycardia was observed in a patient who was managed with 150 mg of oral phenytoin. The patient, in that case, had no prior cardiac history and was on phenytoin for the management of a seizure disorder. All other causes of junctional bradycardia were ruled out when toxic levels of phenytoin were discovered in a serum analysis. The authors concluded that oral phenytoin was the sole cause of the patient’s junctional bradycardia [4]. Finally, a patient who was managed with 200 mg twice daily oral phenytoin for five months following a diagnosis of post-traumatic epilepsy also developed life-threatening junctional bradycardia. On serum analysis, that patient had a serum phenytoin level of 91 µg/mL. The authors assumed that the patient was susceptible to phenytoin toxicity due to his hyponatremia; however, this was inconsistent with the effects seen in the patient’s EKG [5]. Although phenytoin toxicity is most often documented through neurological manifestations, cardiovascular effects should not be ruled out. Although oral phenytoin cardiovascular events are rare, there have been some documented cases showing excessive morbidity in patients on phenytoin.

Oxycodone is a class of opioid that works by binding selectively to Mu opioid receptors. This, in turn, causes the opening of potassium (k+) channels and closes calcium channels (Ca+2), therefore decreasing synaptic transmission. Apart from other pain medications, oxycodone is not thought to have significant adverse effects on cardiovascular function, but as seen with other opioids, it can cause bradycardia and hypotension. One of the rare adverse effects of oxycodone is the release of histamine, which could also induce bradycardia, as it can lead to the vasodilation of arteries [2,6].

Another possibility for the patients' bradycardia presented in this case could be the drug-drug interaction between oxycodone and warfarin, as both drugs are metabolized through the liver. Most opioids have little to no effect on cardiac contractility but drug-drug interactions have been observed when opioids are administered with other drugs, such as warfarin. These drug-drug interactions have led to decreased cardiac function, bradycardia, and vasodilation when used at analgesic doses [2].

## Conclusions

As there is minimal literature supporting the evidence of oxycodone-induced bradycardia due to the lack of studies done, there is a need for larger cohort studies to evaluate the cardiovascular side effects of oxycodone. In this case, we have highlighted persistent, induced bradycardia from a drug-drug interaction of phenytoin and oxycodone. With support from the literature and laboratory results, we infer that the combination of phenytoin and oxycodone were the sole cause of the patient’s persistent bradycardia in this case. From our knowledge, this is the first case of persistent bradycardia with the long-term use of phenytoin and oxycodone reported.
